# Wernicke encephalopathy in a pediatric patient with cannabinoid hyperemesis: A novel case report

**DOI:** 10.1002/jpr3.12109

**Published:** 2024-07-08

**Authors:** Melissa Munroe, Zalan Shah, Aniruddh Setya

**Affiliations:** ^1^ Memorial Healthcare System, Joe DiMaggio Children's Hospital Pembroke Pines Florida USA; ^2^ University of Texas Health Science Center Houston Texas USA; ^3^ Saint Louis University School of Medicine Saint Louis Missouri USA

**Keywords:** adolescent cannabis use, hyperemesis cannabis, thiamine, wernicke

## Abstract

This case report details a novel case of Wernicke encephalopathy (WE) in a 16‐year‐old boy with obesity and chronic cannabis use. Despite the absence of alcohol use disorder, this adolescent developed classic symptoms of WE, challenging the conventional diagnostic framework. Clinical suspicion for WE was supported by typical magnetic resonance imaging findings, low thiamine levels and rapid clinical improvement postintravenous thiamine supplementation. This case underscores the need for heightened clinical vigilance for WE in patients who present with neurologic symptoms who endorse history of persistent emesis, regardless of their history of alcohol use. It also supports the preemptive administration of thiamine in those at risk of deficiency.

**Abbreviations**
CT: computerized tomography
EKG: electrocardiogram
IV: intravenous
MRI: magnetic resonance imaging
PED: pediatric emergency department
QTc: corrected QT interval
WE: Wernicke encephalopathy

## 1 BACKGROUND

Thiamine is an essential cofactor for many metabolic processes and deficiency has the potential to affect all organ systems, especially the nervous system. Wernicke encephalopathy (WE) is a neurologic syndrome characterized by the triad of ophthalmoplegia, cerebellar dysfunction, and altered mental status which results from thiamine (vitamin B1) deficiency.

WE bears a strong association with alcohol misuse wherein individuals often have limited intake of whole foods and in whom thiamine and other micronutrients deficiencies are common. However, WE may also be seen in other states that lead to deficient nutrient intake, absorption or increased consumption. It is well‐recognized that persistent emesis can cause vitamin deficiencies. In fact, there are several case reports of WE in women with hyperemesis gravidarum and even a single case report of WE related to cannabis use in an adult.^1^ However, there are no reports of WE in the pediatric population associated with cannabinoid hyperemesis to date. Here, we discuss the case of a 16‐year‐old diagnosed with WE in the setting of significant cannabis use and with suspicion for cannabinoid hyperemesis syndrome.

## 2 CASE REPORT

A 16‐year‐old male with past medical history of obesity but no other chronic illnesses presented to the pediatric emergency department (PED) with 2 weeks of nausea, emesis, and abdominal pain. He disclosed daily use of inhaled cannabis for several months. He was diagnosed with cannabinoid hyperemesis, treated appropriately with antiemetics and intravenous (IV) fluids and encouraged to discontinue cannabis.

Two months later, he returned to the PED with new complaints of slurred speech, dizziness and visual changes. Initial workup revealed normal computerized tomography (CT) brain, deranged electrolytes, and acute kidney injury presumed secondary to dehydration. Electrocardiogram showed prolonged corrected QT interval (QTc), presumed secondary to ondansetron which he had been taking several times per day for weeks.^2^ He was admitted to the pediatric intensive care unit for IV fluid, cardiac monitoring, and further workup which included an unremarkable CT abdomen and pelvis, as well as lumbar puncture. Post‐IV fluids, his electrolyte abnormalities corrected as did the prolonged QTc. However, emesis persisted and the patient's initial neurologic symptoms progressed to dysarthria, altered mental status, dysmetria, blurred vision and ataxia. Magnetic resonance imaging (MRI) brain revealed a T2 flair in bilateral medial thalami suggestive of cerebral edema.

The presence of ophthalmoplegia, ataxia, and altered mental status, roused suspicion for WE and after extensive workup revealed no other clear etiology for his symptoms, the decision was made to treat presumptively for WE. The patient received IV thiamine three times daily for 3 days, followed by daily for an additional 4 days, and demonstrated marked improvement in symptoms within 48 h. Based on the characteristic bilateral thalamic hyperintensities on MRI, clinical improvement with thiamine supplementation, and low B1 (<2.5 μg/dL), a diagnosis of WE was made.

## 3 DISCUSSION

This case presents a previously unreported phenomenon, the occurrence of WE in a pediatric patient with cannabinoid hyperemesis syndrome.

WE results from thiamine deficiency. Thiamine is an essential cofactor for many metabolic processes and deficiency has the potential to affect all organ systems, especially the nervous system. Thiamine is water soluble and is stored in limited quantities (on average 30−50 mg) primarily in the liver. This vitamin is not produced endogenously and must therefore be ingested in sufficient quantities to meet individual metabolic demands which varies by age and activity level. Most individuals require 0.5−1.5 mg per day.^3^ Therefore, on a thiamine restricted diet, stores are typically depleted in 4−6 weeks. The adolescent male described previously developed symptoms of WE within 2 months of the onset of daily emesis. We believe that his chronic emesis contributed to decreased absorption and possibly intake of thiamine as the patient had begun to engage in restrictive eating practices to decrease likelihood of emesis. It also bears noting that emesis itself may have been a symptom of WE.

There are several unique features of this case which bear noting. Firstly, the patient described experienced significant neurologic deterioration after the administration of dextrose containing IV fluids. Thiamine acts as a cofactor for pyruvate dehydrogenase and α‐ketoglutarate dehydrogenase, two enzymes involved in the metabolism of glucose and production of adenosine triphosphate. In thiamine deficiency, administration of glucose can result in ineffective adenosine triphosphate (ATP) production and accumulation of toxic intermediates which may exacerbate neurologic symptoms. This supports the conventional advice to administer glucose concurrently with thiamine when thiamine deficiency is suspected.

This patient's young age, obesity and absence of chronic alcoholism deviates significantly from the classic patient demographics associated with WE. Atypical presentations and lack of alcohol use disorders have been found to be linked to delayed diagnosis and increased risk of incomplete recovery.^4^ It is critical for clinicians to be aware of diverse etiologies beyond alcohol abuse for WE.^5^, ^6^  This case serves as a reminder to clinicians to maintain a high index of suspicion for WE in patients with persistent vomiting of any etiology.^7^, ^8^

Cannabinoid hyperemesis has become more prevalent in the adolescent population with increasing cannabinoid use. This is exemplified by doubled emergency room visits for cannabinoid hyperemesis in several states postcannabis legalization, signaling a pressing public health concern.^9^

Furthermore, the case described highlights the fact that individuals with elevated body mass index may also have nutrient deficiencies. Contrary to the common perception that obesity precludes vitamin deficiencies, several reports have suggested that diets high in simple sugars and low in thiamine‐rich, whole foods may increase the risk for thiamine depletion.^10^ This is especially relevant given thiamine's critical role in glucose metabolism.

Finally, the significant clinical improvement observed following thiamine supplementation in our patient reinforces the efficacy of this intervention. Given thiamine's safety profile, characterized by its water‐solubility and renal excretion, we advocate for the preemptive administration of thiamine in cases where WE is suspected, even before definitive laboratory confirmation.

The association between cannabinoid hyperemesis and WE in this adolescent patient adds a new dimension to our understanding of WE, emphasizing the diversity of its etiologies beyond chronic alcoholism. It is strongly recommended that clinicians supplement with thiamine immediately when WE is suspected to minimize adverse effects. It is also recommended clinicians consider the shifting trends of substance abuse and diet among the pediatric population and be vigilant for micronutrient deficiencies that may ensue.

## CONFLICT OF INTEREST STATEMENT

The authors declare no conflict of interest.

## ETHICS STATEMENT

We attest that written informed patient consent was obtained for publication of the case details.
